# Fighting against COVID-19: preparedness and implications on clinical practice in primary care in Shenzhen, China

**DOI:** 10.1186/s12875-020-01343-2

**Published:** 2020-12-18

**Authors:** Desiree Man-Sik Tse, Zhuo Li, Ye Lu, Yang Li, Ying Liu, William Chi Wai Wong

**Affiliations:** 1 Department of Family Medicine and Primary Care, Li Ka Shing Faculty of Medicine, The University of Hong Kong, 3/F, Ap Lei Chau Clinic, 161 Main Street, Ap Lei Chau, Hong Kong; 2 Department of Family Medicine and Primary Care, The University of Hong Kong-Shenzhen Hospital, 1 Haiyuan 1st Rd, Futian District, Shenzhen, 518009 Guangdong Province China; 3 Shenzhen Health Capacity Building and Continuing Education Center, National Health Commission, 21 Tian Bei Yi, LuLuohu Qu, Shenzhen Shi, 518041 Guangdong Sheng China

**Keywords:** Primary care, Gatekeeping, Preparedness, COVID-19, Epidemic, China

## Abstract

**Background:**

The new coronavirus pneumonia (NCP) caused by COVID-19 has affected more than 46 million people worldwide. In China, primary care has played a vital role during the COVID-19 outbreak, and it is important to examine the challenges faced by general practitioners (GPs). This study investigated the roles, preparedness and training needs of GPs in China in managing the NCP outbreak. Based on the outcomes of the study, we hope to take lessons and identify how GPs could be supported in delivering their gatekeeping roles and clinical duties in times of infectious disease outbreak.

**Methods:**

An online survey on the official website of Shenzhen Continuing Education Center. It included questions on GPs’ demographics, their awareness of COVID-19 and their preparedness in managing suspected cases of NCP, as well as referrals and their training needs. Conditional multi-variate logistic models were used to investigate the relationships between GPs’ preparedness, situational confidence and anxiety.

**Results:**

GPs’ clinical practice was significantly affected. GPs endeavoured to answer a flood of COVID-19-related enquiries, while undertaking community preventive tasks. In addition to in-person consultations, GP promoted COVID-19 awareness and education through telephone consultations, physical posters and social media. Overall GPs in Shenzhen felt well supported with adequate Personal Protective Equipment (PPE) and resources from secondary care services. Higher levels of self-perceived preparedness (OR = 2.19; 95%CI, 1.04–4.61), lower level of anxiety (OR = 0.56; 95%CI, 0.29–1.09) and fewer perceived family worries (OR = 0.37; 95%CI, 0.12–1.12) were associated with better confidence in coping at work.

**Conclusions:**

Training and supporting GPs while reducing their (and their families’) anxiety increase their confidence in delivering the important roles of gatekeeping in face of major disease outbreaks.

## Background

The World Health Organization (WHO) announced the outbreak of the new coronavirus pneumonia (NCP) caused by COVID-19 as a pandemic on 11th March 2020. Since its emergency in late 2019 from Wuhan, China, COVID-19 has infected more than 46 million people with 1.2 million deaths globally by early November 2020. Thus far, the number of confirmed COVID-19 cases outside China has surpassed those in China. Given the highly contagious nature of the virus with an incubation period of 2–14 days [1–3], the number of cases rose exponentially and hospitals could be overwhelmed within weeks. There are further risks of transmission in healthcare settings, posing a threat to both patients and healthcare providers [4].

Primary care at the forefront in an emerging disease outbreak could play a significant role in gatekeeping and clinical responses - differentiating patients with various respiratory tract symptoms from NCP, making early diagnosis, managing the vulnerable and anxious general public as well as providing care to the otherwise surging, heavy demand in Accident & Emergency Department in hospitals [5, 6]. The number of hospitalisation could be abridged and clinical outcomes significantly improved with an effective community-level triage system when well-trained primary healthcare providers, doctors and nurses alike, treat patients with mild symptoms and refer those with suspected NCP cases to the hospitals, relieving them with more resources to manage the disease outbreak [7].

During a pandemic, absenteeism of healthcare workers, as high as 85% at any time after the outbreak of COVID-19 in Britain [8], is often observed, giving rise to further stress and burden to the healthcare system. Therefore, as suggested by WHO, multi-disciplinary planning and cooperation are vital for effective pandemic responses [9]. It is important to examine the current situation and challenges faced by general practitioners (GPs) during the epidemic.

Since the Healthcare Reforms in 2008, the Ministry of Health in China has issued a directive accrediting the gatekeeper role of their healthcare system towards primary care by redistributing medical resources and patients to the primary care sectors [10]. The tremendous efforts to develop primary care networks have resulted in improvements in access to care in big cities like Shenzhen [11]. Other than village clinics and rural township centres, urban community health care centres (CHCs) have assumed the first port of contact and comprehensive care for patients of different populations [12, 13]. A typical CHC in Shenzhen will cover a population of 30,000–50,000 people with a median of 8 GPs, mostly with 3 years of vocational training [14, 15]. As primary care is a young discipline in China with approximately 15% of GPs working in general practice departments in hospitals, how these primary health workers practised could significantly affect the responses during an epidemic, and demonstrate how resources may affect practice when confronted with similar types of patients [15].

Shenzhen is a major city located in the south of Guangdong Province, the second-worst affected province after Hubei, with a population of 12.53 million. It is the economic powerhouse of China with over 70% of internal economic migrants. With the lockdown of the city on 7th February 2020 along with daily news on the number of new COVID-19 cases and related deaths in the healthcare settings, healthcare providers may suffer significant psychological stress and fears [16], which may further affect their work performance [17, 18]. This study examined if (and how) the outbreak of NCP had impacted on their primary care practice physically and psychologically, bridging any knowledge gap and identify ways to support GPs in fighting against the disease and their role in gatekeeping during an epidemic. It is hypothesised that GPs working in hospitals will have more resources from the authorities to receive more patients and, hence, perform more medical tests, while GPs in working CHCs will rely on resources from the community. As Shenzhen is one of the cities that suffered from the COVID-19 outbreak at an early stage, other regions in the world could take precautions based on its experience.

## Methods

An online survey was posted on the official website operated by the Shenzhen Health Capacity Building and Continuing Education Center (SZHCBCE) under Municipal Health Commission in February 2020. This website is frequently visited by the 3000 GPs who work in Shenzhen. The survey, which took approximately five minutes to complete, was adopted from the severe acute respiratory syndromes (SARS) survey used in Hong Kong in 2004 [19]. The final questionnaire, modified according to the healthcare system in China, was comprised of five dimensions and aimed to investigate: 1) the demographics of the GPs in Shenzhen; 2) their awareness of COVID-19; 3) their preparedness in managing suspected cases of NCP; 4) primary and secondary care interface; and 5) their training needs.

The main outcome measures were changes of clinical behaviour and practices during the epidemic, and anxiety level of primary care doctors. Based on the previous study that 75% of doctors would change their clinical practice or behaviours in an emerging infection outbreak [19], a sample size of 345 responses was estimated for a 90% power and standard error of 0.05 with an attrition rate of 20%. The survey was pilot-tested amongst 16 local GPs, and some items were modified based on the clinical and medical practice of GPs working in Shenzhen. For example, perceived confidence, preparedness, and anxiety were each accessed with a self-reported item, with fixed-choice questions.

### Statistical analysis

Descriptive analyses were performed to study the demographic factors related to change of clinical practice and preparedness of the respondents. *Chi*-square statistics were employed to compare distributions of changes between GPs working in hospitals and CHCs/private clinics. Multi-variate logistic regression models were used to investigate the relationships between GPs’ preparedness, situational confidence, and anxiety; with likelihood ratios and Hosmer & Lemeshow goodness-of-fit test to assess model fitness. All statistical analyses were performed with Statistical Package for the Social Sciences (SPSS) 22.0, with two-tailed statistical tests and a significance level of 0.05.

The study protocol was approved by the Medical Ethics Committee in The University of Hong Kong-Shenzhen Hospital (hkuszh2020010).

## Results

A total of 382 questionnaires were received. However, twenty-one with incomplete data were removed, leaving the final sample of 361. Table 1 presents the demographics of the respondents. GPs working in hospitals and CHCs/private clinics participated in this study were not different in age, gender, or marital status.

**Table 1:** Demographic information of respondents

	Count (%)
Age (Mean ± SD)	39.27 ± 7.77
Gender	
Female	198 (54.8%)
Male	163 (45.2%)
Year of graduation	
1970–1979	3 (0.9%)
1980–1989	13 (4.1%)
1990–1999	79 (24.8%)
2000–2009	125 (39.2%)
2010–2019	99 (31.0%)
Marital status	
Married	320 (88.6%)
Single	41 (11.4%)
Type of medical institutions	
CHC/Private clinic	307 (85.0%)
Hospital	54 (15.0%)

### Impacts of outbreak on clinical practice

The outbreak of COVID-19 affected almost every GP’s clinical practice (95.6%)(Table 2). Despite 83.1% of respondents reported that fewer patients had visited their clinics during the epidemic, only 13.6% of them had adhered to their usual practice while over a quarter (27.4%) of the GPs extended their work hours to cope with the outbreak. More efforts were spent to ensure the patients entering the clinics with a face mask (82.0%) or without fever (78.9%). Few clinics (9.1%) were closed.

**Table 2:** Changes in clinical practices or behaviour amongst the GPs in Shenzhen during the NCP outbreak

	Hospital (*n* = 54)	CHC/Private clinic (*n* = 307)	
Clinical practice being affected by NCP	50 (92.6%)	298 (97.1%)	
Change in service in clinic			
No change in service demand	4 (7.4%)	9 (2.9%)	
Lower demand for services (seeing fewer patients)	44 (81.5%)	256 (83.4%)	
Higher demand for services (seeing more patients)	3 (5.6%)	4 (1.3%)	
Others	3 (5.6%)	38 (12.4%)	
Change in clinical practice			
Increase office hours	13 (24.1%)	86 (28.1%)	
Shorten patient consultation time	19 (35.2%)	93 (30.3%)	
Ask patients to go to other clinics or hospitals	40 (74.1%)	190 (61.9%)	
Cancel or change regular, non-acute appointments	50 (92.6%)	120 (39.1%)	
Close the clinic	4 (7.4%)	29 (9.5%)	
Community visits to educate and detect suspected NCP cases	11 (20.4%)	238 (77.5%)	
Follow up on suspected NCP cases	142 (46.3%)	13 (24.1%)	***
Handle phone/online enquiries	12 (22.2%)	183 (59.6%)	***
Others	9 (16.7%)	90 (29.4%)	
Change in medical practice			
Insist every patient to wear a mask	45 (83.3%)	251 (81.5%)	
Screening/measure temperature as a routine procedure	40 (74.1%)	245 (79.8%)	
Increase distance between the doctor and the patient	37 (68.5%)	189 (61.6%)	
Avoid physical examination	23 (42.6%)	130 (42.3%)	
Order more blood tests and chest X-ray	35 (64.8%)	68 (22.1%)	***
Order less blood tests and chest X-ray	5 (9.3%)	26 (8.5%)	
More referrals to A&E Department	24 (44.4%)	108 (35.2%)	
Advise them not to travel to those affected areas	36 (66.7%)	227 (73.9%)	
Others	1 (1.9%)	9 (2.9%)	

*** *p*-value < .001 for comparison between hospital and CHC/private clinics using Chi-squared test

GPs’ main duties were re-arranged to handle general or COVID-19-related enquiries (54.0%) and prevent community outbreak (15.8%). Compared to those GPs working in hospitals, more community GPs working in CHCs or private clinics were assigned to answer telephone/online enquiries (*χ*^2^(1) < 0.001); and follow-up on suspicious cases (*χ*^2^(1) < 0.001). In contrast, more X-ray imaging and blood tests were performed by hospital GPs (*χ*^2^(1) < 0.001) (Table 2).

### Preparedness of GPs

Table 3 presents how well the clinics and GPs have prepared during the epidemic. In order to plan and equip healthcare workers in fighting against the virus, nearly all (98.6%) clinics conducted seminars and training, with the majority giving three or more talks since the outbreak (88.6%), particularly in CHCs or private clinics (90.5%). In addition, masks and PPE were more readily in the hospital setting and our hospital colleagues were likely to handle NCP cases with more psychological support. Nearly all GPs (99.2%) still perceived a dire need to enrich their knowledge, particularly in handling of suspected cases (74.2%); preventive measures during home visits (67.3%); use of personal protective equipment (65.1%); handling diagnostic samples (57.3%); and, providing counselling patients’ psychological needs (80.6%). Education was not only provided for healthcare workers but also for the general public with 98.9% of the GPs educated their patients during consultation (83.9%); via social media or online platforms (80.9%); over telephone (61.2%); and, by sharing relevant information as posters or leaflets in the clinic (54.0%).

**Table 3:** Preparedness of GPs for the NCP outbreak in Shenzhen

	Hospital (*n* = 54)	CHC/Private clinic (*n =* 307)
Number of training sessions for NCP		
> =3	42 (77.8%)	278 (90.5%)
2	8 (14.8%)	16 (5.2%)
1	3 (5.6%)	9 (2.9%)
0	1 (1.9%)	4 (1.3%)
Protective materials provided in medical provisions		
N95 face masks	13 (24.1%)	75 (24.4%)
Surgical masks	50 (92.6%)	298 (97.1%)
Personal protective equipment (PPE)	14 (25.9%)	106 (34.5%)
Familiar with referral procedures	53 (98.1%)	307 (100.0%)
Knowledge gaps identified by GPs		
Use of PPE	37 (68.5%)	198 (64.5%)
Diagnostic sampling	33 (61.1%)	174 (56.7%)
Preventive measures in suspected NCP cases	39 (72.2%)	204 (66.4%)
Handling suspected NCP cases	36 (66.7%)	232 (75.6%)
Providing psychological support for suspected NCP cases	40 (74.1%)	251 (81.8%)
Referral procedures	25 (46.3%)	164 (53.4%)
Encountered suspected NCP cases	20 (37.0%)	92 (30.0%)

The clinics also provided the protective gears such as surgical face masks (96.4%), N-95 face masks (24.4%), and PPE, including disposable surgical gowns and gloves (33.2%). Six GPs (1.7%) working in hospitals with a shortage of mask supply stated that they were making their own such as facial screen or protective clothing. This interesting finding was reflected in the item accessing their overall response on whether their clinics provided enough support to protect the staff at work, in which only 15% of the hospital GPs believed they were “sufficient”.

Other than providing treatment, two-third of the GPs (63.7%) had been referring suspected patients to other clinics or hospitals. All, but one, GPs thought they were familiar with the referral pathway, which overall considered to be “smooth, efficient and timely” (97.8%). Despite only a third (31.0%) of them having encountered the likely cases, the majority (86.7%) were confident in diagnosing and handling these cases.

### Situational confidence and anxiety of GP

While approximately half (44.3%) of the GPs reported being anxious, fearful or, having sleep difficulties due to the outbreak of NCP, most of their families (75.9%) were worried about them providing frontline care. Two conditional multi-variate logistic regression models were performed on preparedness, confidence in handling cases and anxiety of GPs and their families. An overall higher levels of self-perceived preparedness (OR = 2.19; 95%CI, 1.04–4.61); lower levels of anxiety (OR = 0.56; 95%CI, 0.29–1.09); and lower levels of perceived family worries (OR = 0.37; 95%CI, 0.12–1.12) were associated with better confidence in coping at work during the epidemic. Another model also found an overall higher anxiety perceived by GPs was associated with not being confident (OR = 0.55; 95%CI, 0.29–1.05) and more worries from their family (OR = 6.92; 95%CI, 3.58–13.35).

## Discussion

The current study found that GPs in China, particularly in big cities such as Shenzhen, were overall well-equipped and supported during the NCP outbreak, even though their clinical practice and behaviours were invariably affected since the outbreak. The anxiety level among GPs in Shenzhen was moderate, partly related to their well-preparedness and confidence in handling the current situation. Furthermore, there were few differences in the clinical behaviour or practice between the GPs working in hospitals vs. the community, except that hospital GPs tended to order more investigations while community GPs had contacted patients more through telephone or online communication tools. These findings, along with that indicating “insufficient” PPE provided at work, shows that community GPs might have fewer resources during the COVID-19 epidemic [13]. It might also further support that the general public in China preferred to consult hospital GPs, as they perceived the community health care service as inferior [20]. However, the increase in X-ray imaging and blood tests in the hospital setting could also be explained by patients’ requests and/or their availability at hospitals. Moreover, it is known that NCP is more accurately diagnosed with a reverse-transcription polymerase chain reaction (RT-PCR) test or a chest computed tomography (CT) which are not generally available in the primary care setting. More education is required to bridge the knowledge gap in the diagnosis of NCP, emphasising the discrepancies between X-ray and CT scan in detecting the disease.

Unlike Wuhan, where COVID-19 had emerged and taken the city by surprise, Shenzhen was much better planned for emergency response measures and had in place disease control systems for a possible outbreak [21]. In response to the epidemic, apart from the special arrangements in fever triage and detecting suspected cases involving the primary care team, there is also an Infectious Disease Epidemic Plan (IDEP) to facilitate the management and containment of the virus [16]. Consistent with the IDEP, GPs in our study have changed their practice, in which elective procedures and ambulatory clinics have been suspended. Instead, telephone and online consultations, made available by the Directives from Guangdong Health Bureau at the end of 2019, were adopted. The increase of virtual consultations also shows that communication, rather than investigations, was preferred when the latter was not readily available. Furthermore, GPs, as civil servants working for the Chinese government, were assigned to community visits as well as public health education. Plans were implemented to educate healthcare providers in preventing potential transmission of COVID-19 at the clinical and community levels.

Despite disruptions in their clinical practice, our study showed that GPs’ overall confidence level in fighting against the virus was high, which was positively correlated with a sense of preparedness and negatively correlated with anxiety and worries from family. GPs’ confidence in their capability to complete assigned duties, in addition to managing cases in an epidemic, are perhaps the results of their preparedness after pre-crisis education and training programmes [22, 23]. In contrast with our previous research on the SARS epidemic in the neighbouring city, Hong Kong, the GPs in Shenzhen were generally supported and trained, particularly for infectious disease outbreaks, for example, they were familiar with the referral and management pathways for suspected cases [15]. This bidirectional relationship between the situational confidence and anxiety among GPs identified in our study further emphasises the importance of preparedness in combating infectious disease outbreaks. Owing to the joint efforts of hospitals, clinics, patients, and the Chinese government’s “most ambitious agile and aggressive disease containment effort in history”, including travelling restrictions and lockdown of the cities [21]; the spread of the virus was seemingly contained with the number of new COVID-19 cases peaking at mid-March 2020. Preparedness in primary care is closely related to these healthcare providers’ confidence and anxiety level at work during an epidemic, other countries are recommended to act promptly to fight against the pandemic.

### Strengths and limitations

Our sample size of 361 GPs represented 10–15% of the GPs registered in Shenzhen, the response rate was impressive for an online survey given that the survey was only posted on the website and there was no particular incentive. However, it may not be a representative sample since it was not randomly selected, which was not possible given the timeframe and resources available to us. Arguably the respondents would be a keener group of doctors or more affected by the pandemic, such that they were more motivated to complete the survey. Nonetheless, this study had the power to pick up major changes among the healthcare providers during the outbreak. In other words, any significant findings observed would be of importance. Secondly, the data collected was self-reported, which could introduce self-selected bias, particularly in assessing family worries about the GPs providing frontline service.

### Implications for practice

After months of efforts, the number of new COVID-19 cases has dropped since early March 2020 in China. Shenzhen, being the worst affected city in Guangdong, has also resumed work at the end of March 2020. As of 13th November 2020, the number of diagnosed cases stayed at 471 in the city. Many GPs continues to provide online healthcare services, which were widely adopted to reduce the burden on hospitals by providing medical diagnosis and treatment online. Even though the Directives from Guangdong Health Bureau changed the regulations in 2019 to allow online consultations, the public did not show much enthusiasm. Not until the COVID-19 pandemic, the number of online consultations had increased by 15-fold. Primary care continues to play a crucial role in transforming the healthcare system in China. Post COVID-19, online medical services would become more widely accepted, and careful evaluation and more resources are needed to prepare the healthcare workers to this new form of doctor-patient relationship in the future.

According to a report by the WHO and World Bank, the healthcare system in China was “hospital-centric, fragmented, and volume driven” [24, 25]. Following the Healthcare Reform, the medical institutions in China had developed various clinical pathways to multidisciplinary care plans to be implemented at local levels; yet healthcare systems and consumers in the country are still heavily weighted towards specialist care, and primary care was not perceived as a desirable career path by healthcare workers. This is partly due to the misconception that primary healthcare was seen as a service for the poor, and not using such service would be regarded as a desirable social status in some Chinese communities [12]. However, primary care has proven its critical gatekeeping and health educator roles during the pandemic, providing online medical services as well as performing. Public health tasks. With well-preparedness and confidence to handle the situation, GPs have made a significant contribution to halt the COVID-19 outbreak. It is believed that Shenzhen has curbed the disease with the primary care actively involved at the earlier stage. In preparing for a disease outbreak, a large and diverse country would require multi-level of complexity, taking into consideration the multifaceted structures, planning and response processes in the nation [26].

## Conclusions

The current study showed how the GPs in China, particularly in big cities such as Shenzhen, practised during the NCP outbreak. We found that preparedness in the healthcare settings is closely related to healthcare providers’ confidence and their anxiety level at work during an epidemic, other countries are recommended to act promptly to fight against the pandemic.

## Data Availability

The datasets used and/or analysed during the current study are available from the corresponding author on reasonable request.

## Abbreviations

WHO: World Health Organisation
NCP: New coronavirus pneumonia
GPs: General practitioners
CHCs: Community health centres
SZHCBCE: Shenzhen Health Capacity Building and Continuing Education Center
SARS: Severe acute respiratory syndromes
SPSS: Statistical Package for the Social Sciences
RT-PCR: Reverse-Transcription Polymerised Chain Reaction
CT: Computed tomography
IDEP: Infectious Disease Epidemic Plan
